# Characterization of prostanoid pathway and the control of its activity by the eyestalk optic ganglion in the female giant freshwater prawn, *Macrobrachium rosenbergii*

**DOI:** 10.1016/j.heliyon.2021.e05898

**Published:** 2021-01-30

**Authors:** Tipsuda Thongbuakaew, Chanudporn Sumpownon, Attakorn Engsusophon, Napamanee Kornthong, Charoonroj Chotwiwatthanakun, Prasert Meeratana, Prasert Sobhon

**Affiliations:** a School of Medicine, Walailak University, Nakhon Si Thammarat, 80161, Thailand; b Department of Anatomy, Faculty of Science, Mahidol University, Bangkok, 10400, Thailand; c College of Medicine, Rangsit University, Pathum Thani, 12000, Thailand; d Chulabhorn International College of Medicine, Thammasat University, Pathum Thani, 12120, Thailand; e Center of Excellence for Shrimp Molecular Biology and Biotechnology (CENTEX Shrimp), Faculty of Science, Mahidol University, Bangkok, 10400, Thailand; f Nakhonsawan Campus, Mahidol University, Nakhonsawan, 60000, Thailand; g Faculty of Allied Health Science, Burapha University, Chon Buri, 34315, Thailand

**Keywords:** Prostanoid pathway, Reproduction, Eyestalk ablated, *Macrobrachium rosenbergii*

## Abstract

The giant freshwater prawn, *Macrobrachium rosenbergii*, is an economically valuable species that are distributed throughout the Asia-Pacific region. With the natural population declining due to overfishing, aquaculture of this species is deemed necessary. Hence, it is essential to understand the mechanisms regulating reproduction in order to increase their production. Prostaglandins (PGs) play an important role in reproduction in most vertebrates and several invertebrates. It has been proposed that crustaceans have PGs but the prostanoids pathway in the giant freshwater prawn is still unclear. In this study, we identified 25 prostanoid-related genes involved in the biosynthesis of active prostanoids in *M. rosenbergii* using *in silico* searches of transcriptome data. Comparative analysis of encoded proteins for the *MroPGES2* gene with other species was performed to confirm their evolutionary conservation. Gene expression analysis revealed the correlation of *MroPGES2* gene expression level with the progress of ovarian development. Eyestalk ablation increased the expression level of *MroPGES2* gene compared to intact groups during the ovary maturation stages. Collectively, this study confirmed the existence of prostanoids in the giant freshwater prawn, as well as characterizing key gene *MroPGES2* associated with the prostanoid pathway. We propose that *MroPGES2* may play an important role in *M. rosenbergii* ovarian maturation and its expression is under the inhibitory control from the eyestalk optic ganglion hormones. Identification of genes in prostanoid pathway and their expressions enables future functional studies to be performed, which may lead to applications in the aquaculture of this species.

## 1 Introduction

*Macrobrachium rosenbergii*, the giant freshwater prawn, has been highly valued as food in the tropical countries of Asia for domestic consumption and export (Hossain and Das, 2010; New and Nair, 2012). The demand for this prawn is rapidly increasing and necessitates the production of this species by aquaculture. Understanding the processes that control gonadal maturation and gamete production are key to success in aquaculture of this species. Reproduction of the giant freshwater prawn as well as other crustaceans is a highly complex process that is controlled by neuroendocrine factors (Nagaraju, 2011; Subramoniam, 2011). Manipulation actors of some key factors may help to increase their reproduction by aquaculture (Okumura, 2004).

Prostaglandins (PGs) play important roles in several essential physiological processes including reproduction in most animals (Rowley et al., 2005; Wimuttisuk et al., 2013). The biosynthesis of prostanoids from their precursors occurs through the action of key enzymes, especially cyclooxygenase (COX) and specific terminal prostanoids synthases (Smith, 2008). PGs have been reported to play a role in stimulation of the ovarian maturation including vitellogenesis and spawning in many decapod crustaceans (Tahara and Yano, 2004; Rowley et al., 2005; Sumpownon et al., 2015). The ovarian level of PGs is highly correlated with vitellogenic stages of the ovarian cycle in the crayfish, *Procambarus paeninsulanus* (Spaziani et al., 1993), the kuruma prawn, *Marsupenaeus japonicus* (Tahara and Yano, 2004) and the giant freshwater, *M. rosenbergii* (Sumpownon et al., 2015). Administration of PGs increased the ovarian-somatic index and oocyte diameter in the freshwater crab, *Oziotelphusa senex senex* in dose-dependent manner (Reddy et al., 2004). Moreover, putative genes related to prostanoid biosynthesis are reported in the water flea, *Daphnia pulex* (Heckmann et al., 2008) and also in the black tiger shrimp, *Penaeus monodon* (Wimuttisuk et al., 2013). Although the presence of prostanoids and their precursors is well-established in most crustaceans, prostanoid biosynthesis pathway, key enzymes in this process, as well as their functions on reproduction in crustaceans still remain unclear. Furthermore, no previous work has been done on the prostanoid pathway in female *M. rosenbergii*. Thus, the purposes of our studies are 1) to identity the complete set of enzymes involved in prostanoid biosynthesis pathway in the female *M. rosenbergii* through transcriptome analysis; 2) to characterize and investigate the tissue expressions of the key enzymes, namely, prostaglandin E synthase 2 (PGES2), representing the downstream or rate-limiting step enzyme in the production of PGE2 the major PG that is proven active in ovarian maturation (Sumpownon et al., 2015); and 3) to investigate whether the expressions of this enzyme is under the control of eyestalk optic ganglion hormones, especially gonad-inhibiting hormone (GIH) (Diwan, 2005; Nagaraju, 2011; Uawisetwathana et al., 2011). These studies will provide basic knowledge concerning the prostanoid biosynthesis pathway that controls the reproductive processes, particularly the ovarian maturation in this decapod crustacean, which may be applied to the increase of prawn reproduction.

## 2 Materials and methods

### 2.1 Sequence annotation, gene mining, and protein prediction

All relevant transcriptome data for CNS and ovarian tissues of female *M. rosenbergii* was obtained from NCBI Sequence Read Archive (SRA) database (accession number: SRP049917) reported by Suwansa-ard et al. (2015) (Suwansa-ard et al., 2015). Briefly, transcripts were selected and compared against the databases of NR, NT, Swiss-Prot, KEGG, COG, and GO, using BLAST and BLAST2GO software, with an E-value threshold of 1e-6. Relative abundance of all transcripts among different tissues was estimated by SOAP software version 2.21 (Suwansa-ard et al., 2015). Homologs of prostanoid-related genes were identified by tBLASTn searches against known prostanoid-related genes reported in previous studies, using the CLC Main Workbench Version 7.7 (CLC Bio-Qaigen, AsiaPac, Taiwan). All hits were analyzed manually with their homologous proteins from various species and the presence of conserved motifs. Analysis of protein similarity was performed by protein alignment using MUSCLE (https://www.ebi.ac.uk/Tools/msa/muscle/) (Edgar, 2004). Prediction of conserved protein domains were performed by NCBI conserved domain database (Marchler-Bauer et al., 2017) and InterPro (https://www.ebi.ac.uk/interpro) (Finn et al., 2017). Protein precursors were analyzed for their evolutionary relationship with other known homologous proteins, and illustrated by phylogenetic trees using the MEGA X program using Neighbor-joining estimation (1000 bootstraps) (Kumar et al., 2018). Illustrations were indicated by the genus, species name, and NCBI accession number.

### 2.2 Animals and tissue collection

All methods were carried out in accordance with relevant guidelines and regulations for using animals. All the experimental procedures presented in this work were approved by the Animal Care and Use Committee of Walailak University, National Research Council of Thailand (NRCT), Protocol No. 005/2019.

Live mature female *M. rosenbergii* were obtained from a Phran Nok market, Bangkok, Thailand. They were then acclimatized in culture tanks at the Faculty of Science, Mahidol University. These included mature female prawns with different stages of the ovarian cycle [stages 1–4, as described previously (Meeratana and Sobhon, 2007)]; 20 prawns/stage; n = 80, with average weight of 30–40 g, and mature male prawns, n = 20, with average weight of 150–200 g. After 24 h in culture tanks, the animals were anesthetized by immersion in ice-cold water for 5 min before sacrifice. The eyestalks, brains, thoracic ganglia, abdominal ganglia, ovaries, testes, hepatopancreases, hematopoietic tissues, guts, hearts, and muscles were collected and immediately frozen in liquid nitrogen, then stored at -80 °C until preparation of total RNA.

### 2.3 Total RNA extraction

Frozen tissues were individually homogenized and total RNA extracted with TRIzol reagent (Thermo Fisher Scientific, MA, USA) following the manufacturer's protocol in combination with a DNaseI (Thermo Fisher Scientific, MA, USA) treatment to eliminate potential genomic DNA contamination. The quantity and quality of RNA samples were measured using spectrophotometry (NanoDrop 1000; Thermo Fisher Scientific, DE, USA). Total RNA of each tissue was pooled and dried separately.

### 2.4 Tissue expression of *MroPGES2* by RT-PCR

Two micrograms of total RNA of each tissue were used for cDNA synthesis. Complementary DNA (cDNA) was generated by reverse transcription of total RNA using RevertAid RT kit (Thermo Scientific, USA) following the manufacturer's protocol. Gene-specific primers for *MroPGES2* gene were designed using the Primer-BLAST program (https://www.ncbi.nlm.nih.gov/tools/primer-blast) (Ye et al., 2012) (Table 1). PCR was carried out using the PCR SuperMix (Thermo Fisher Scientific, MA, USA) following a routine protocol optimized for the primers. *Beta-actin* gene was used as a positive control, while the negative control was non-RT cDNA. PCR products were analyzed by agarose gel electrophoresis. The amplicons of expected size were extracted by QIAquick gel extraction kit (QIAGEN, Hilden, Germany) and subcloned into the pDrive vector (QIAGEN, Hilden, Germany). The plasmids were purified by QIAprep Spin Miniprep Kit (QIAGEN, Hilden, Germany), and sequenced by Macrogen (Macrogen Ltd., Seoul, South Korea). The obtained sequences were analyzed using multiple bioinformatics tools including BLAST against the NCBI GenBank database, and the putative amino acid sequence was deduced by using Expasy bioinformatics tool (http://web.expasy.org/translate/) (Artimo et al., 2012).

**Table 1:** Gene-specific primers used for tissue expression and quantitative real-time PCR.

Genes	Techniques	Forward primer (5′-3′)	Reverse primer (5′-3′)	Size (bp)
*MroPGES2*	RT-PCR	TGACTCGGGCACAAACAAGA	TGAGGTCCTCGAAAGCATCAC	560
	qPCR	AGATGAAAGGAAGTGGCGCA	GCTGCTGCACCAACATAAACA	180
*Beta-actin*	RT-PCR/qPCR	GCAGGAGATGACCACCGAAA	GGATGCCGCAGGATTCCATA	152

### 2.5 Expression of *MroPGES2* in developing oocytes by *in situ* hybridization (ISH)

The spatial distribution of PGESs expression in the ovarian tissue sections was detected by ISH. Briefly, the ovaries were dissected out and fixed in fresh 4% paraformadehyde fixative in 0.1M PBS, pH 7.4 at 4 °C overnight. Then the tissues were processed by routine paraffin method. Paraffin embedded blocks were sectioned at 6 μm thickness. *MroPGES2* gene was PCR-amplified with M13 primers using the plasmid containing the *MroPGES2* gene as a template (forward; 5′ GTAAAACGACGGCCAGT 3′ and reverse primer; 5′ AACAGCTATGACCATG 3′). The PCR products were processed through separation and extraction using QIAquick Gel Extraction Kit (QIAGEN, Hilden, Germany) and used as a template for riboprobe synthesis using a DIG-oligonucleotide labeling kit (Roche, Germany). The *in situ* hybridization was performed following the previous described protocol (Thongbuakaew et al., 2019). The stained sections were observed and photographed under Nikon E600 microscope equipped with a DXM1200F digital microscope (Nikon, Tokyo, Japan).

### 2.6 Determinations of the expression of *MroPGES2* in each ovarian stage of intact and eyestalk ablated female prawns by real-time PCR

Mature female prawns at stage 1 of ovarian cycle (80 prawns/group) were separated into 2 groups; intact and eyestalk ablated. They were acclimatized in a culture tank for 2–3 days before performing experiment. Each ablated female prawn had one of its eyestalks removed close to the base with a scalpel. The incision spot was closed with an electric cauterizer and antibiotic pomades. The ovarian tissues of intact and eyestalk ablated female prawns in each stage were collected throughout the entire ovarian cycle, immediately frozen in liquid nitrogen, and stored at -80 °C until use. Total RNA was extracted from the tissues as described previously in section 2.3 and converted to cDNA as described previously in sections 2.4. All primers used for the analysis are shown in Table 1. *Beta-actin* gene was used as the internal control. The qPCR was performed using GeneRead qPCR SYBR green mastermix (Qiagen, Hilden, Germany) following the manufacturer's protocol. The qPCR amplification was performed on the Applied Biosystems 7500 Fast Real-Time PCR (Applied Biosystems, CA, USA) following a protocol optimized for *MroPGES2* gene with dissociation curve analysis. Transcripts were quantified using a standard curve method (Larionov et al., 2005). Standard curves for *MroPGES2* and *beta-actin* were generated by 10-fold serial dilutions of known concentrations of the plasmids containing the target transcripts. The detection range, linearity, and real-time PCR amplification efficiency of each primer pair were checked before continuing with sample analysis. The qPCR reaction efficiency was calculated from the standard curve, which ranged from 90 to 100%. Expression of *beta-actin* was used as the internal reference to correct for differences in reverse transcription efficiency and template quantity. All standards and experimental samples were run in duplicate. The results were analyzed using the ABI 7500 Fast Software v1.4. Transcript levels of *MroPGES2* in the ovary were normalized by the level of *beta-actin* and the data was expressed as relative mRNA levels.

### 2.7 Statistical analysis

Data were expressed as a mean ± S.D, analyzed and compared using a one-way analysis of variance (ANOVA), followed by a Tukey's post hoc multiple comparison. The probability value less than 0.05 (p < 0.05) indicated a significant difference.

## 3 Results

### 3.1 Gene mining and identification of prostanoid-related genes

**Figure 1:**
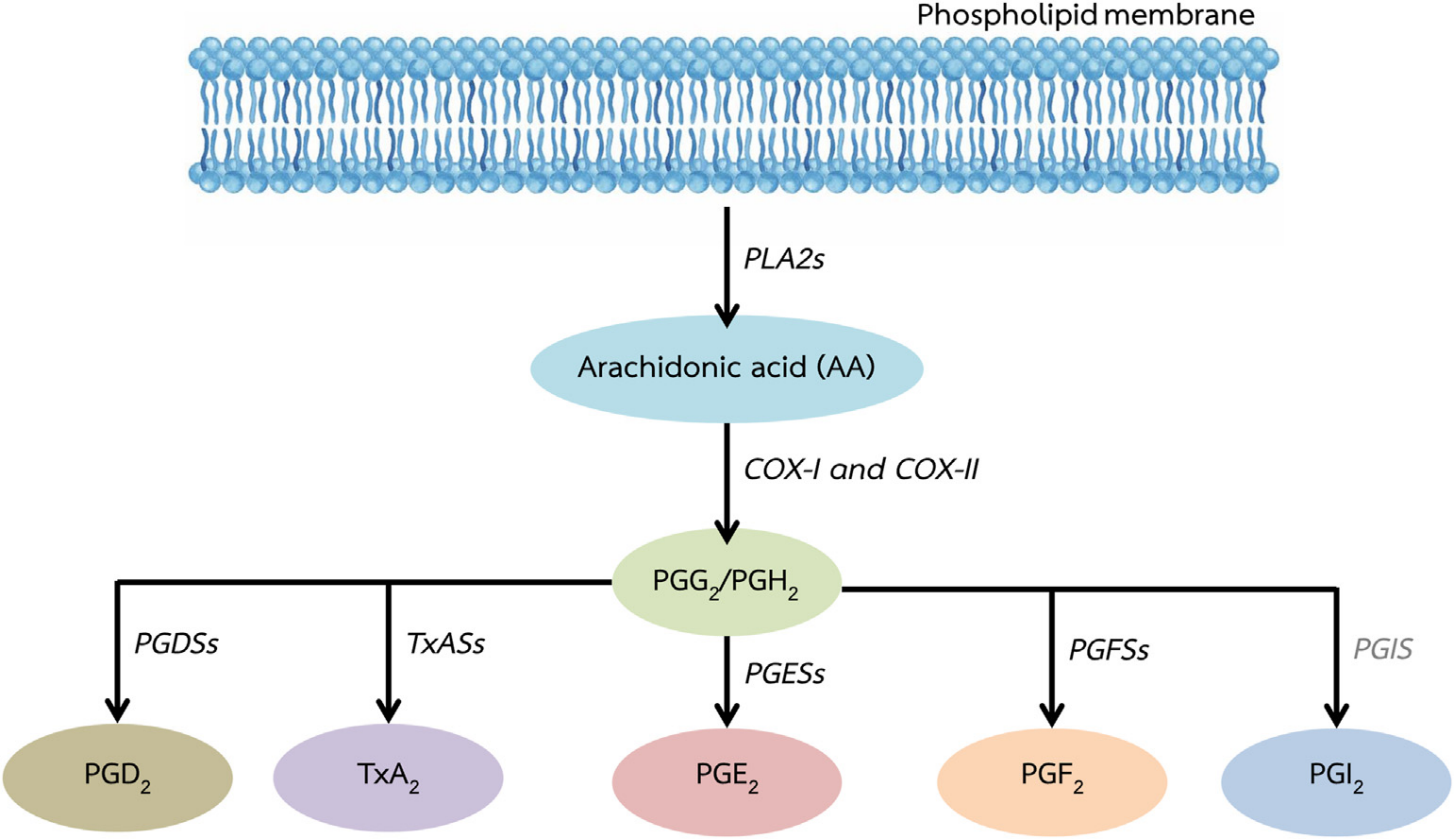
Schematic diagram showing the putative pathway for prostanoid biosynthesis in the *Macrobrachium rosenbergii*. We identified phospholipase A2s (PLA2s), cyclooxygenase 1 (COX-I), cyclooxygenase 2 (COX-II), prostaglandin D synthases (PGDSs), prostaglandin E synthases (PGESs), prostaglandin F synthases (PGFSs), and thromboxane A synthases. Prostaglandin I synthase (PGIS) was not found in the *M. rosenbergii* (gray letter).

We used a *de novo* assembled transcriptome for *M. rosenbergii* to identify prostanoid-related genes. We found 25 transcripts encoding prostanoid-related genes, including members of the enzymes involved in prostanoid pathway. Sequence and annotation information is provided in Table 2. Moreover, Figure 1 illustrates the biosynthesis pathway position of the enzyme genes identified that are involved in the biosynthesis of active prostanoids in *M. rosenbergii.*

**Table 2:** Summary, including BLAST annotation, of genes found in *M. rosenbergii* involved in the prostanoid pathway.

Prostanoid-related genes	Transcript	ES (TPM value)	CNS (TPM value)	Ov (TPM value)	BLAST hit and species	E-value	Accession numbers
Cytosolic phospholipase A2 (cPLA2)	Unigene19244_All Unigene7080_All	5.95	14.72	0.38	Cytosolic phospholipase A2 [*Penaeus monodon*]	0	AFJ11391.1
Secreted phospholipase A2 (sPLA2)	Unigene6650_All	10.69	36.54	1.98	Group 3 secretory phospholipase A2 [*Stegodyphus mimosarum*]	4E-42	KFM61512.1
Phospholipase C delta (PLCd)	Unigene7737_All	6.99	15.59	1.86	Phospholipase C delta, putative [*Ixodes scapularis*]	2E-94	XP_002410916.1
Phospholipase C beta (PLCb) isoform 1	Contig19390_MrCNS	4.68	13.09	0.91	Phospholipase C beta 1 [*Bombyx mori*]	4E-165	AAD32609.1
Phospholipase C beta (PLCb) isoform 2	Unigene36025_All	12.24	1.17	0.34	Phospholipid phospholipase C beta isoform [*Homarus americanus*]	0	AAD32609.1
Phospholipase C gamma (PLCg)	Unigene57328_All	0.34	0.67	8.65	Phospholipase C gamma [*Bombyx mori*]	3E-119	NP_001165394.1
Cyclooxygenase 1 (COX1)	Unigene20053_All Unigene27866_All	12.98	15.21	0.69	Cyclooxygenase [*Halocaridina rubra*]	0	ALG96666.1
Cyclooxygenase 2 (COX2)	Unigene22278_All	6.10	13.39	24.22	Cyclooxygenase [*Halocaridina rubra*]	0	ALG96666.1
Glutathione-dependent prostaglandin D synthase (gPGDS) isoform 1	Unigene3543_All	10.62	12.97	3.86	Glutathione-dependent prostaglandin D synthase [*Penaeus monodon*]	5E-95	AFJ11393.1
Glutathione-dependent prostaglandin D synthase (gPGDS) isoform 2	Unigene3451_All	21.71	56.72	5.44	Glutathione-dependent prostaglandin D synthase [*Penaeus monodon*]	3E-27	AFJ11393.1
Hematopoietic prostaglandin D synthase (hPGDS) isoform 1	Unigene20336_All	27.70	374.43	55.93	Hematopoietic prostaglandin D synthase [*Penaeus monodon*]	3E-74	AFJ11392.1
Hematopoietic prostaglandin D synthase (hPGDS) isoform 2	Unigene19478_All	9.08	5.93	31.86	Hematopoietic prostaglandin D synthase [*Penaeus monodon*]	2E-52	AFJ11392.1
Hematopoietic prostaglandin D synthase (hPGDS) isoform 3	Unigene19813_All	3.29	0.43	4.15	Hematopoietic prostaglandin D synthase [*Penaeus monodon*]	9E-22	AFJ11392.1
Cytosolic prostaglandin E synthase (cPGES)	Unigene7084_All	35.98	67.13	126.43	Cytosolic prostaglandin E synthase [*Penaeus monodon*]	2E-67	AFJ11394.1
Microsomal prostaglandin E synthase (mPGES) isoform 1	Unigene17352_All	9.06	33.92	4.91	Microsomal prostaglandin E synthase [*Penaeus monodon*]	1E-57	AFJ11395.1
Microsomal prostaglandin E synthase (mPGES) isoform 2	Unigene49139_All	1.85	1.45	4.56	Microsomal prostaglandin E synthase [*Penaeus monodon*]	1E-29	AFJ11395.1
Prostaglandin E synthase 2 (PGES2)	Unigene3911_All, Unigene19799_All	5.13	5.88	71.86	Prostaglandin E synthase 2 [*Penaeus monodon*]	0	AFJ11396.1
Prostaglandin F synthase 1 (PGFS1)	Unigene26298_All	25.35	38.56	299.44	Prostaglandin F synthase [*Penaeus monodon*]	0	AFJ11397.2
Prostaglandin F synthase 2 (PGFS2)	Unigene23331_All	1.80	2.97	14.37	Prostaglandin F synthase-like [*Aplysia californica*]	2E-103	XP_005089972.1
Thromboxane A synthase isoform 1	Unigene48062_All	1.05	1.94	113.63	Thromboxane-A synthase-like [*Oreochromis niloticus*]	1E-84	XP_013127958.1
Thromboxane A synthase isoform 2	Unigene45852_All	0.83	4.37	0.34	Thromboxane A synthase-like protein [*Daphnia pulex*]	1E-52	EFX87565.1
Thromboxane A synthase isoform 3	Unigene46960_All	0.00	2.96	0.53	Thromboxane A synthase [*Penaeus monodon*]	6E-21	AFJ11398.1
15-hydroxyprostaglandindehydrogenase	Contig677_MrCNS	1.89	6.91	0.36	15-hydroxyprostaglandin dehydrogenase-like [*Lingula anatina*]	2E-64	XP_013396931.1
Aldo-keto reductase	Unigene299_All	5.74	7.44	37.30	Aldo-keto reductase 1 [*Coptotermes gestroi*]	4E-113	AMJ21949.1
Carbonyl reductase	Unigene10725_All	13.45	23.40	68.45	Carbonyl reductase 1 [*Zootermopsis nevadensis*]	2E-121	KDR03826.1

### 3.2 Characterization of MroPGES2

**Figure 2:**
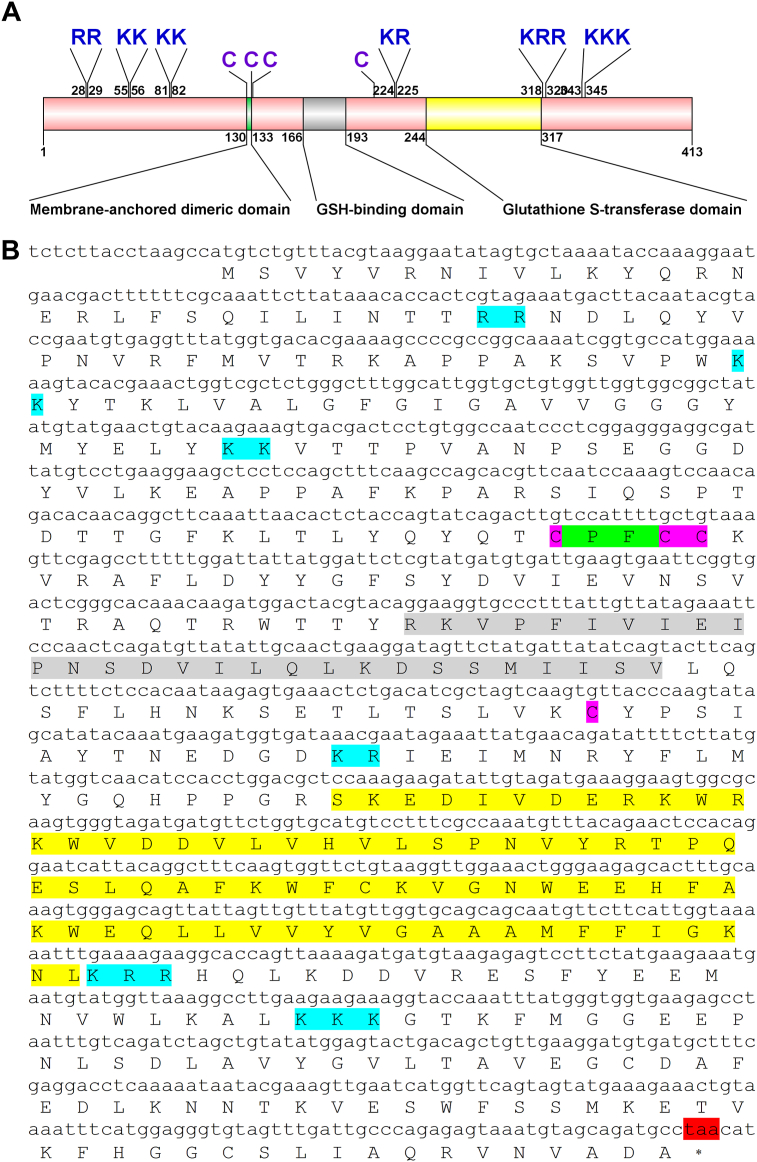
Molecular characterization of the *Macrobrachium rosenbergii* prostaglandin E synthase 2 (MroPGES2) precursor. (A)A schematic diagram showing the structure of MroPGES2 precursor, and (B)corresponding nucleotide and amino acid sequences. MroPGES2 ORF contains the predicted membrane-anchored dimeric domain (highlighted in light green), GSH-binding domain (highlighted in gray), and glutathione S-transferase domain (highlighted in yellow), cleavage sites (highlighted in light blue), and conserved cysteines (highlighted in purple). Asterisk indicates the stop codon (highlighted in red).

**Figure 3:**
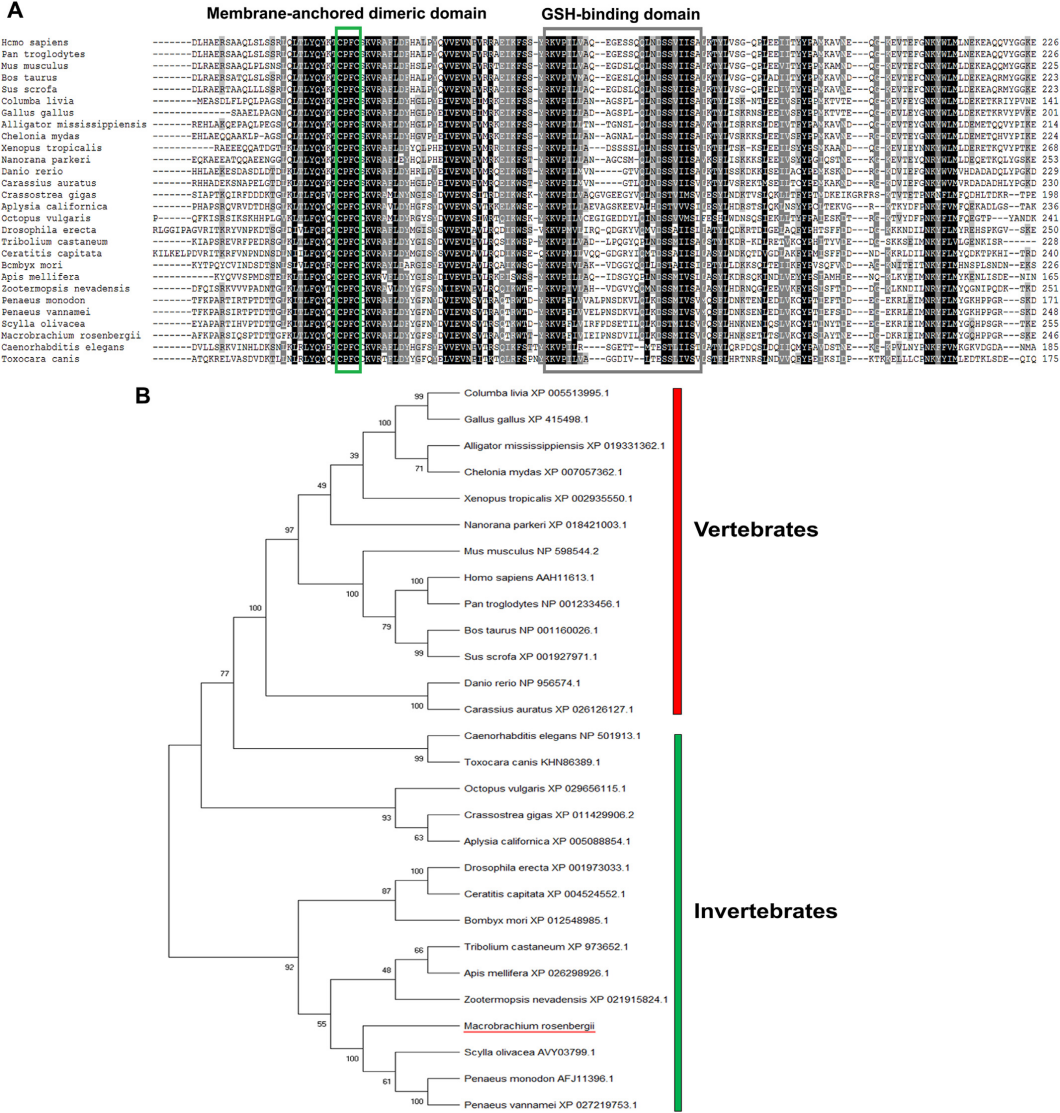
Multiple sequence alignment and phylogenetic tree of *Macrobrachium rosenbergii* PGES2 precursor and homologs from other species. (A) Alignment of PGES2 demonstrates conservation among species within key domains, membrane-anchored dimeric domain (Cys-x-x-Cys) (green box) and GSH-binding domain (gray box). Conserved amino acids are shown in black shading while similar amino acids are shown with grey shading. (B) Phylogenetic tree of PGES2 precursors constructed based on Neighbor-joining analysis with 1000 replicates bootstrap. Bootstrap values are indicated for each branch divergence. Amino acid sequences and their accession number are shown in S1.

*MroPGES2* transcript encoding a full-length protein is composed of 413 aa (Figure 2A). MroPGES2 contains conserved membrane-anchored dimeric domain (Cys-x-x-Cys) and GSH-binding domain (Figure 2A-B). At C-terminal, MroPGES2 also contains glutathione S-transferase domain (Figure 2A-B). Moreover, MroPGES2 contains 4 conserved cysteine residues predicted to form 2 disulfide bridges (Figure 2A-B). Alignment between MroPGES2 with other species homologs demonstrates conservation within the key motifs (Figure 3A). The MroPGES2 clusters with homologs of crustaceans and insects and is clearly distinguished from vertebrate homologs (Figure 3B).

### 3.3 Tissue expression of *MroPGES2* and spatial distribution of *MroPGES2* in developing oocytes

**Figure 4:**
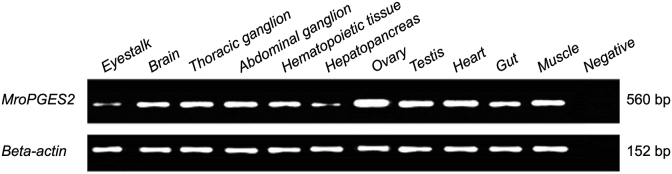
Agarose gel showing tissue-specific expression of *Macrobrachium rosenbergii PGES2* gene using RT-PCR with specific primers. The *beta-actin* gene was used as a positive control. Negative control was performed without cDNA. The expected amplicon size is shown in base pairs (bp). Full images of gels are shown in S2.

*MroPGES2* expression was observed in all investigated tissues including, eyestalk, brain, thoracic ganglion, abdominal ganglion, hematopoietic tissue, hepatopancreas, ovary, muscle, heart, gut and testis but more abundantly expressed in ovary. However, signal was weak level in eyestalk and hepatopancreas (Figure 4).

**Figure 5:**
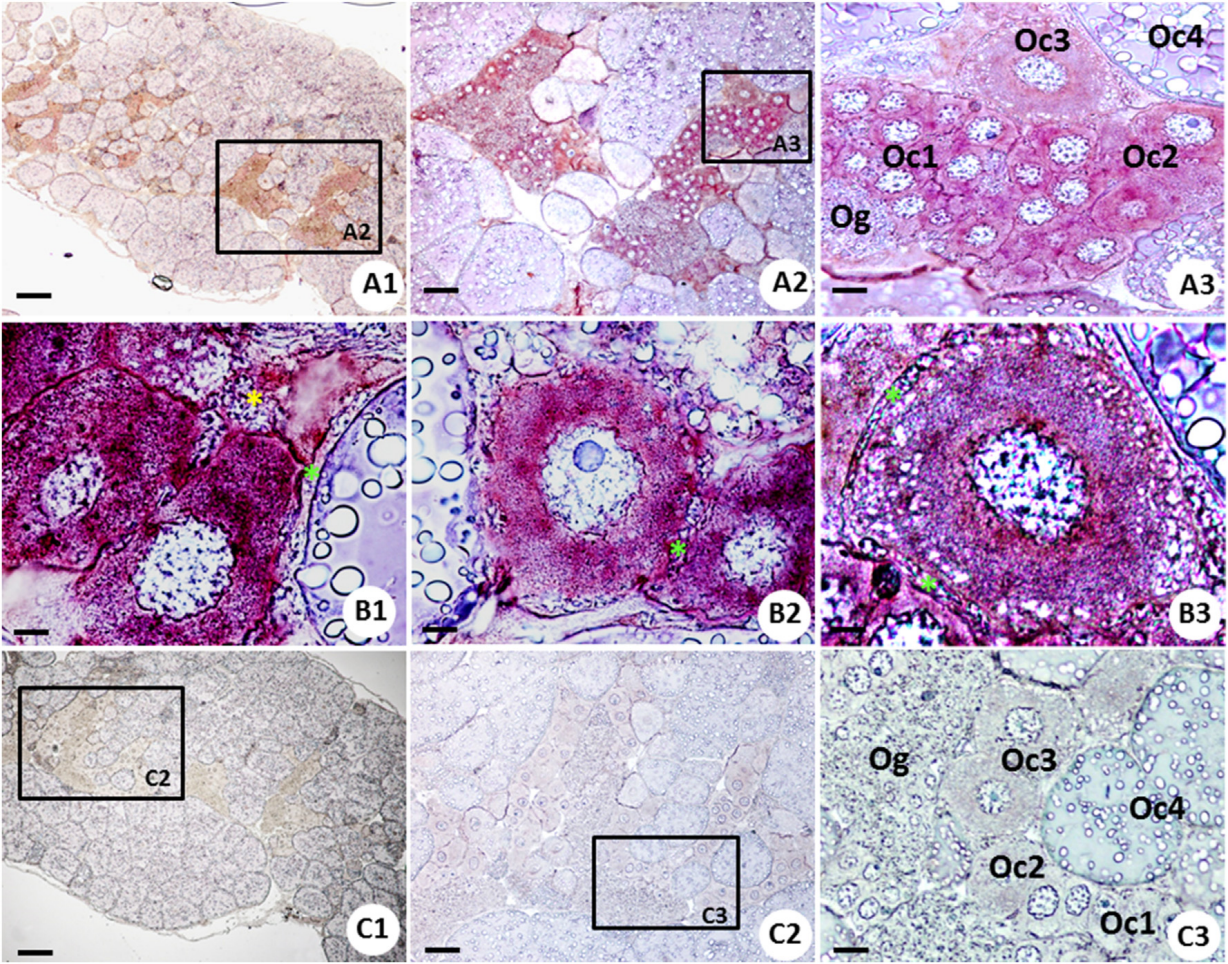
*In situ* hybridization localization of *MroPGES2* mRNA transcripts in the ovary of the *Macrobrachium rosenbergii*. (A1-A3) Localized expression of *MroPGES2* in the cytoplasm of oogonia (Og), previtellogenic oocytes (Oc1 and Oc2) and vitellogenic oocytes (Oc3). (B1–B3) High magnification showing positive staining in the cytoplasm of Oc1, Oc2, Oc3, respectively, and follicular cell type 1 (green asterisks) and type 2 (yellow asterisks). (C1–C3) Negative control micrograph using a DIG-labeled sense-strand *MroPGES2* riboprobes showing no positive signal in the ovary. Scale bars represent 250 μm (A1, C1), 100 μm (A2, C2), 25 μm (A3, C3), 10 μm (B1–B3). Abbreviations: Oogonia (Og); Oocyte 1 (Oc1); Oocyte 2 (Oc 2); Oocyte 3 (Oc3); Oocyte 4 (Oc4).

Next, we determined expression and spatial distribution of *MroPGES2* in developing oocytes by ISH, and found positive signal in the cytoplasm of oogonia (Og), previtellogenic (including Oc1 and Oc2) and early vitellogenic (Oc3) oocytes (Figure 5A1-A3), and follicular cell type 1 (Fc1) and 2 (Fc2) (Figure 5B1-B3). Strong positive staining was observed in previtellogenic oocytes, whereas the Og and Oc3 showed less intense staining (Figure 5A3). No gene expression was observed within the late vitellogenic oocyte (Oc4) and mature oocytes (mOc). As well no signal was detected in the negative control in which *MroPGES2* sense riboprobes were used (Figure 5C1-C3).

### 3.4 Expression of *MroPGES2* in each ovarian stage of intact and eyestalk ablated female prawns

**Figure 6:**
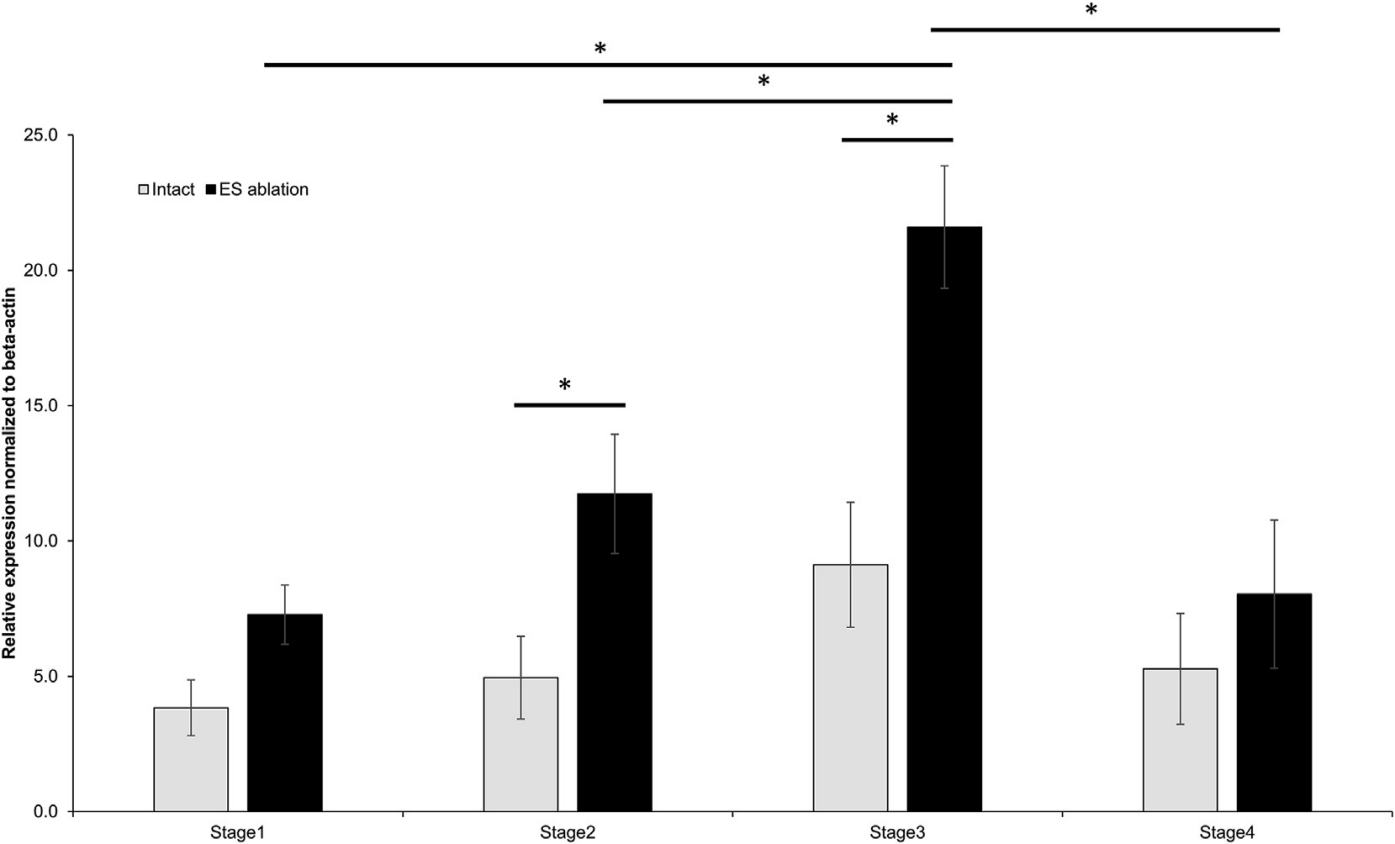
Relative gene expression levels of *MroPGES2* in different ovarian stages using quantitative real-time-PCR. Data were normalized against beta-actin and represented as mean ± S.D. ∗ Indicates statistically different (p < 0.05).

We further analyzed the relative gene expression in the ovarian tissues of intact and eyestalk ablated female prawns during gonad maturation by real-time PCR (Figure 6). Results revealed that the expression of *MroPGES2* gradually increased from stage 1 to stage 3 of ovarian development and decreased at mature stage (stage 4). *MroPGES2* gene expression was markedly increased in the stage 3 of ovarian development. Importantly, eyestalk ablated female prawns showed higher expression level of *MroPGES2* at all stages, and markedly at stages 2 and 3, of the ovarian cycle when compared to intact female prawns.

## 4 Discussion

Our present study has proven the existence of prostanoid pathway in the giant freshwater prawn, *M. rosenbergii*, as well as genes involved in the biosynthesis of active prostanoids. The occurrences of PGs biosynthesis in invertebrate animals have been established in insects (Stanley, 2006; Stanley and Kim, 2014) and aquatic invertebrates (Rowley et al., 2005). Fully constructed eicosanoid biosynthesis pathway in the *Daphnia* has been reported based on bioinformatic and transcriptomic evidences, which revealed nine prostanoid biosynthetic genes (Heckmann et al., 2008). Moreover, COX and PGES have been identified in amphipod crustaceans, *Gammarus spp.* and *Caprella spp.* (Varvas et al., 2009; Hansen et al., 2014). The putative prostanoid pathway has been shown in the black tiger shrimp, *P. monodon*, which contains only nine prostanoid biosynthesis genes (Wimuttisuk et al., 2013). Our study demonstrated that *M. rosenbergii* has the same types of prostaglandin synthase enzymes and prostanoid pathway as those found in vertebrates and other species.

PGE2 is the most common prostanoid, which is converted from PGH2 by PGES enzyme, and it plays an important role in a variety of actions, including reproductive functions in vertebrates (Sun et al., 2006; Milatovic et al., 2011) and invertebrates (Stanley, 2006; Stanley and Kim, 2014; Sumpownon et al., 2015; Duangprom et al., 2018). In the present study, the sequence of *MroPGES2* in the *M. rosenbergii* was identified and its deduced amino acid sequence was predicted. Sequence comparison by amino acid alignment of PGES2 proteins from several species indicated that PGES2 are relatively conserved, especially at the key motifs, which comprised of the membrane-anchored dimeric domain (Cys-x-x-Cys) and GSH-binding motif. Based on a human PGES2 model (Sjögren et al., 2013), silkworm *Bombyx mori* (Yamamoto et al., 2013), the penaeid shrimp, *P. monodon* (Wimuttisuk et al., 2013), and lepidopteran insect, *Spodoptera exigua* (Ahmed et al., 2018), these domains are responsible for catalyzing the isomerization of PGH2 to PGE2. Phylogenetic comparison of the MroPGES2 precursors with other sequences revealed that MroPGES2 clusters with invertebrates and most closely related to other crustaceans. However, invertebrate PGESs forms distinct clades from vertebrates due to low heme-binding affinity when compared with vertebrate PGESs (Hansen et al., 2014). This suggested that MroPGES2 is responsible for PGE2 biosynthesis in *M. rosenbergii* and shares a common ancestor with vertebrates and other species.

By RT-PCR we found the expression of *MroPGES2* mRNA in all organs studied but the expression was strongest in ovary. Investigation of spatial gene expression by ISH indicated that *MroPGES2* was expressed in various developing oocytes and follicular cells but not in late vitellogenic oocyte (Oc4). This pattern of expression implied that in the *M. rosenbergii*, *MroPGES2* might be involved in controlling early oocyte development and ovarian maturation. Similarly, *PGES2* mRNA was localized in various tissues and in the oocytes and follicular cells of the mud crab, *Scylla olivacea*, and that PGES may be involved with oocyte development in crab (Duangprom et al., 2018). As well, the presence of PGES2 in the ovary of the penaeid shrimp, *P. monodon*, suggested its possible involvement in the oocyte development (Preechaphol et al., 2010). In the freshwater crab, *O. senex senex*, PGE2 was detected in many tissues, including ovaries with greater gene expression during vitellogenesis (Reddy et al., 2004). Moreover, PGE2 was detected by immunoperoxidase at relatively higher level in the Oc1 and Oc2 than other stages of ooytes of the giant freshwater prawn, *M. rosenbergii* (Sumpownon et al., 2015), and in the early stage of oocytes of the kuruma prawn, *M. japonicus* by use immunoenzyme assay (Tahara and Yano, 2004). These findings suggested PGE2 plays specific role in oocyte development especially in vitellogenesis (Tahara and Yano, 2004; Sumpownon et al., 2015). Moreover, PGE2 also plays a role in the mediating oocyte maturation in the zebrafish (Lister and Van Der Kraak, 2008). Taken together, these findings suggest a possible and important role of PGES2 and PGE2 in controlling oocyte development. Their roles in other processes of female reproduction such as spawning in crustaceans has also been suggested (Tahara and Yano, 2004; Sumpownon et al., 2015).

By using qPCR, we found that the amount of *MroPGES2* gene expression increased as the ovary developed and decreased when it reached mature stage. This is consistent with the previous study in the giant freshwater prawn, *M. rosenbergii*, which reported that PGE2 level in the ovaries was high during early stages of the ovarian cycle and subsided in late stages (Sumpownon et al., 2015). As well, the amount of PGES2 gradually increased during ovary development in the mud crab, *S. olivacea* (Duangprom et al., 2018). The concentration of PGE2 was strongly correlated with ovarian maturation in the crayfish, *P. paeninsulanus* (Spaziani et al., 1993, 1995), the freshwater edible crab, *O. senex senex* (Reddy et al., 2004), the kuruma prawns, *P. japonicas* (Tahara and Yano, 2004), and the penaeid shrimp, *P. Monodon* (Preechaphol et al., 2010). Furthermore, in eyestalk-ablated prawns the expression level of *MroPGES2* was increased in early stages of ovarian maturation when compared to intact prawns, and the increases were most notable during stages 2 and 3. As it was well known that the eyestalk optic ganglia secrete many important hormones involved in ovarian maturation, including GIH which inhibits the synthesis of vitellogenin (Treerattrakool et al., 2008; Nagaraju, 2011; Uawisetwathana et al., 2011). Removing eyestalk induced early ovarian maturation in many decapods (Okumura et al., 2006; Okumura, 2007; Fernandez and Radhakrishnan, 2016) by removing GIH as demonstrated in the pink shrimp, *Penaeus notialis* (Rosas et al., 1993). Moreover, eyestalk ablation significantly increased vitellogenin expression level in the hepatopancreas and ovary resulting in acceleration of ovary maturation by removing inhibitory hormone in the oriental river prawn, *Macrobrachium* nipponense (Bai et al., 2015). Known reproductive genes involved in ovarian maturation were dramatically increased after removing eyestalk in the black tiger shrimp, *P. monodon* (Uawisetwathana et al., 2011). Furthermore, RNA interference of GIH reduced the GIH transcript level resulting in stimulation of ovarian maturation in *P. monodon* (Treerattrakool et al., 2008). Thus, we suggested that *MroPGES2* expression might be controlled by GIH from the eyestalk.

## 5 Conclusion

In conclusion, a putative prostanoid pathway and prostanoid-related genes in the *M. rosenbergii* had been demonstrated, and they showed conservation with crustaceans and other species. MroPGES2 and PGE2 expressions in ovaries were high during early stages of the ovarian cycle as well as in early oocytes, but subsided when the ovaries reached maturity and the oocytes became fully developed. Thus, MroPGES2 and PGE2 might be involved in oocyte development and vitellogenesis. Eyestalk ablation shortened the period of ovarian maturation and significantly enhanced the levels of *MroPGES2* expression in the ovaries compared to those of the unablated prawns. Thus, the inhibitory eyestalk hormones, possibly GIH might control the expression of *MroPGES2*. This knowledge on prostanoid pathway and roles of MroPGES2 and PGE2 may be applied to increase the production of this species in aquaculture.

## Declarations

### Author contribution statement

T. Thongbuakaew: Conceived and designed the experiments; Performed the experiments; Analyzed and interpreted the data; Contributed reagents, materials, analysis tools or data; Wrote the paper.

C. Sumpownon: Conceived and designed the experiments; Performed the experiments; Analyzed and interpreted the data; Wrote the paper.

P. Sobhon: Conceived and designed the experiments; Contributed reagents, materials, analysis tools or data.

A. Engsusophon and N. Kornthong: Performed the experiments.

C. Chotwiwatthanakun and P. Meeratana: Contributed reagents, materials, analysis tools or data.

### Funding statement

T. Thongbuakaew was supported by the Individual Research Grant, Walailak University, Thailand (WU-IRG-62-001) and the Research Grant for New Scholar, Thailand Research Fund (TRF) (MRG6280007).

### Data availability statement

Data will be made available on request.

### Declaration of interests statement

The authors declare no conflict of interest.

### Additional information

No additional information is available for this paper.

## Appendix A Supplementary data

S1 S1

S1 S1
