# Supplementary figures and images for: Characterization of prostanoid pathway and the control of its activity by the eyestalk optic ganglion in the female giant freshwater prawn, Macrobrachium rosenbergii
Source: Heliyon. 2021 Jan 30;7(1):e05898. doi: 10.1016/j.heliyon.2021.e05898 (PMC7851786; doi:10.1016/j.heliyon.2021.e05898)

MroPGES2 gel

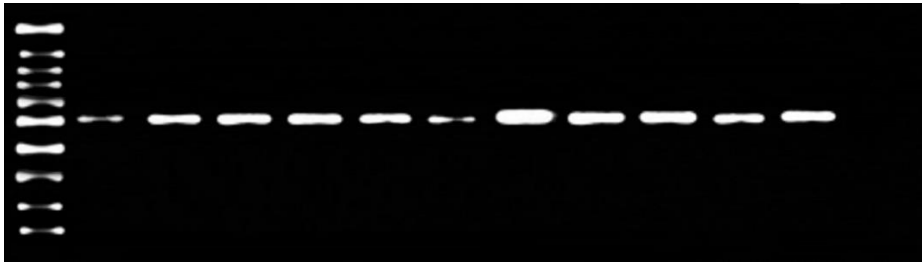

Beta-actin gel

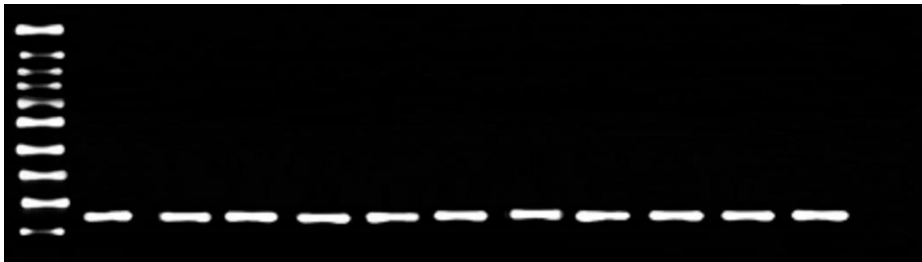

Supplement: S1 [file mmc2.pdf]
